# A Non-Invasive Scoring System to Differential Diagnosis of Clear Cell Renal Cell Carcinoma (ccRCC) From Renal Angiomyolipoma Without Visible Fat (RAML-wvf) Based on CT Features

**DOI:** 10.3389/fonc.2021.633034

**Published:** 2021-04-23

**Authors:** Xiao-Jie Wang, Bai-Qiang Qu, Jia-Ping Zhou, Qiao-Mei Zhou, Yuan-Fei Lu, Yao Pan, Jian-Xia Xu, You-You Miu, Hong-Qing Wang, Ri-Sheng Yu

**Affiliations:** ^1^ Department of Radiology, Second Affiliated Hospital, Zhejiang University School of Medicine, Hangzhou, China; ^2^ Department of Radiology, Wenling Hospital of Traditional Chinese Medicine, Taizhou, China; ^3^ Department of Radiology, The Second Affiliated Hospital of Zhejiang Chinese Medical University, Hangzhou, China; ^4^ Department of Ultrasonic, Wenzhou Central Hospital, Wenzhou, China; ^5^ Department of Radiology, First Affiliated Hospital of Wenzhou Medical University, Wenzhou, China

**Keywords:** clear cell renal cell carcinoma, renal angiomyolipoma without visible fat, computed tomography, scoring system, differential diagnoses

## Abstract

**Background:**

Renal angiomyolipoma without visible fat (RAML-wvf) and clear cell renal cell carcinoma (ccRCC) have many overlapping features on imaging, which poses a challenge to radiologists. This study aimed to create a scoring system to distinguish ccRCC from RAML-wvf using computed tomography imaging.

**Methods:**

A total of 202 patients from 2011 to 2019 that were confirmed by pathology with ccRCC (n=123) or RAML (n=79) were retrospectively analyzed by dividing them randomly into a training cohort (n=142) and a validation cohort (n=60). A model was established using logistic regression and weighted to be a scoring system. ROC, AUC, cut-off point, and calibration analyses were performed. The scoring system was divided into three ranges for convenience in clinical evaluations, and the diagnostic probability of ccRCC was calculated.

**Results:**

Four independent risk factors are included in the system: 1) presence of a pseudocapsule, 2) a heterogeneous tumor parenchyma in pre-enhancement scanning, 3) a non-high CT attenuation in pre-enhancement scanning, and 4) a heterogeneous enhancement in CMP. The prediction accuracy had an ROC of 0.978 (95% CI, 0.956–0.999; P=0.011), similar to the primary model (ROC, 0.977; 95% CI, 0.954–1.000; P=0.012). A sensitivity of 91.4% and a specificity of 93.9% were achieved using 4.5 points as the cutoff value. Validation showed a good result (ROC, 0.922; 95% CI, 0.854–0.991, P=0.035). The number of patients with ccRCC in the three ranges (0 to <2 points; 2–4 points; >4 to ≤11 points) significantly increased with increasing scores.

**Conclusion:**

This scoring system is convenient for distinguishing between ccRCC and RAML-wvf using four computed tomography features.

## Introduction

Approximately 75% of renal cell carcinomas are clear cell renal carcinomas (ccRCC) (1), the most common presentation of which is a renal mass. However, no malignant tumor among the renal masses accounts for more than 20%, and renal angiomyolipoma (RAML) is one of the most common benign cell types (2). RAML can often be diagnosed easily, due to macroscopic fat tissue within the tumor that can be detected by imaging. However, about 5% of RAML has insufficient fat for identification using conventional imaging modalities; these are regarded as RAML without visible fat (RAML-wvf) (3, 4). RAML-wvf mimics ccRCC on imaging due to the absence of fat, and is often diagnosed incorrectly (5). This presents a difficult challenge to radiologists and clinicians; since misdiagnosis might cause harm for patients, diagnosis is typically confirmed through biopsy or surgery. For distinguishing between RCC and RAML-wvf, the tumor texture, unenhanced computed tomography (CT) density, enhancement pattern, chemical shift parameter, sonographic features, and other imaging indexes were useful in previous reports (6–10). Most reports were based on qualitative analysis of imaging features because of the low incidence of RAML-wvf

CT is the preferred imaging method for evaluating renal mass in clinical settings (11). Previous studies have attempted to identify useful strategies based on CT imaging to differentiate ccRCC from RAML-wvf A hyperattenuating mass on unenhanced CT with homogeneous enhancement pattern on enhanced CT is highly suggestive of RAML-wvf (12–15). Investigators also tried detecting hidden fat tissue within the renal masses by counting negative-attenuation pixels using CT scans, thin-section (2–5 mm) scanning, and histogram analysis (16–18). However, these strategies are either too subjective or too time-consuming. Therefore, some quantitative methods have been reported recently, such as CT texture analysis (19, 20), machine learning-based texture analysis (11), and a CT-based radiomics nomogram (21). However, these methods might not be sufficiently convenient for clinical application.

Therefore, we aimed to identify characteristic CT features that could be used to distinguish patients with ccRCC from those with RAML-wvf weighted scores were assigned to the resulting model to make it more concise and convenient for use in clinical practice.

## Materials and Methods

### Study Population

**Figure 1:**
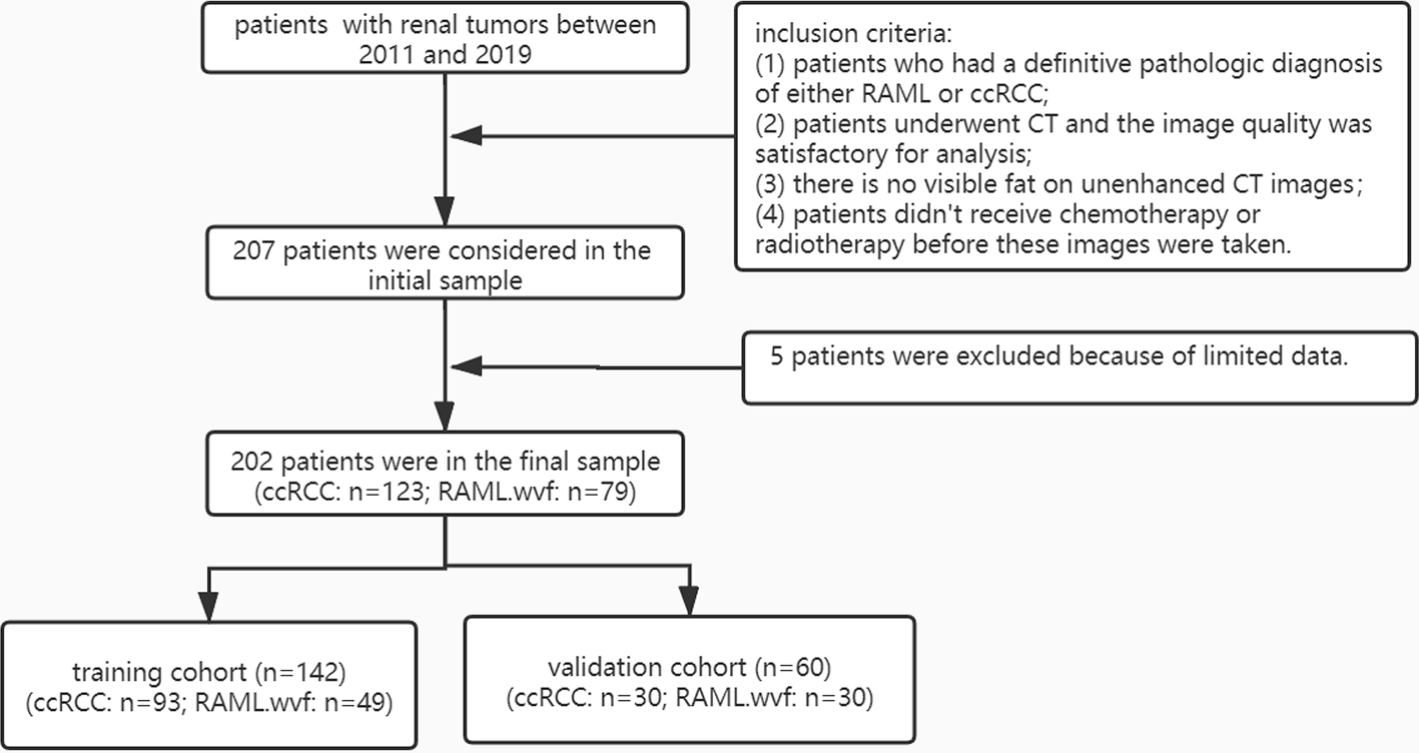
Patient flow diagram.

A total of 202 patients from 2011 to 2019 that were confirmed with ccRCC (n = 123) or RAML (n = 79) by pathology were retrospectively analyzed. The inclusion criteria were: 1) patients who had a definitive pathologic diagnosis of either RAML or ccRCC, 2) patients underwent CT and the image quality was satisfactory for analysis, 3) there was no visible fat on unenhanced CT images, and 4) patients did not receive chemotherapy or radiotherapy before the images were taken. There were five patients excluded because of limited data. The 202 patients were divided randomly into a training cohort (n = 142) and a validation cohort (n = 60) (**Figure 1**).

### Acquisition of Images

CT examinations were performed with multidetector CT (SOMATOM Definition Flash; Siemens Healthcare and LightSpeed 16; GE Healthcare). The scanning parameters were 120 kVp tube voltage, 220 mA tube current, slice thickness, and a 5-mm slice interval. Enhanced scanning was performed in three phases, including the post-contrast corticomedullary phase (CMP) (delay 30 s), post-contrast nephrographic phase (NP) (delay 90 s), and post-contrast excretory phase (EP) (delay 180 s).

### Analysis of Images

CT images were evaluated independently by two abdominal radiologists who were blinded to the pathology results. The observed variables of CT features included the tumor number (single or multiple), location, contour (regular or irregular), and edge (clear or blurred), the existence of special findings (calcification, necrotic or cystic, pseudocapsule, wedge-shape sign, round tumor-kidney interface), features of the tumor parenchyma in pre-enhancement scanning, features of the total tumor in different scanning phases, and the enhancement pattern.

The tumor locations were classified into four patterns: A) the whole mass located in the renal parenchyma, B) the proportion of the mass that highlights the outline of the kidney <50%, C) the proportion of the mass that highlights the outline of the kidney >50%, and D) the mass grew into the renal medulla. A pseudocapsule was defined as an unenhanced arc area between the lesion and renal parenchyma. A wedge-shaped sign indicated that the tumor was triangular and pointing to the renal hilum.

The tumor CT attenuation in pre-enhancement scanning was categorized as high or not-high density compared with that of renal parenchyma (a difference > 5 HU). Heterogeneity was defined as the difference between the highest and lowest attenuations being more than 30% of the highest value. Heterogeneous parenchyma in pre-enhancement scanning was considered when the parenchyma mass that could be enhanced was mixed. The enhanced scanning ratio 1 (ESR 1) was defined as the CT attenuation of the lesion minus the renal parenchyma in the CMP; ESR 2 was minus the aorta in the NP. The lesions significantly enhanced in CMP were classified as either “fast-in, fast-out” (the lesion quickly cleared in NP), “fast-in, slow-out” (the lesion was cleared in EP), or “persistent enhancement” (the lesion was still enhanced in EP).

Radiologists carefully outlined a 20-mm^2^ region of interest (ROI) to include as much tissue mass as possible, avoiding necrotic or cystic areas when the CT attenuation of the tumor parenchyma was obtained. The ROI was determined at least twice, and the average was taken before obtaining the final CT attenuation. In addition, other clinical data (e.g., age, sex) were collected for all cases.

### Statistical Analysis

Continuous variables were calculated as median with range (M-R), and categorical variables as the frequency with percentage. Data of the training cohort were used to establish the scoring system. The same variables between patients with RAML-wvf and with ccRCC were compared using the Student t-test for continuous variables and the chi-square or Fisher’s exact test for categorical variables. Variables that were significant in univariate analysis were obtained to a logistic regression model after confirming there was no multicollinearity. For the training of an integer-based distinguishing scoring system, we decided to use the method described by Ben Ayed et al. (22). We first used the following formula to get the initial value: β/β_min_ (β, regression coefficient of each variable; β_min_, minimum value of regression coefficient), which was rounded to the nearest integer to get the final score of each CT feature. The total score was calculated by summing the individual score corresponding to the related variables. The performance of the predicting model was evaluated by discrimination and calibration metrics. The receiver operating characteristic (ROC) was used to assess the discriminatory power of the model, and the Hosmer-Lemeshow goodness-of-fit test evaluated the calibration (23). A comparison among ROC of different models has been performed using the Delong nonparametric method (24). Further validation was performed using data from another 60 independent patients.

All the data were analyzed by SPSS version 25.0 software (IBM Crop, Aromonk, NY), except ROC comparison performed by MedCale statistical software, version 19.0 (MedCale Software bvba), P < 0.05 was defined as statistically significant.

## Results

### Characteristics of the Study Patients

Differences in clinical and CT characteristics between patients with RAML-wvf and ccRCC are presented in **Table 1**. Sex, necrosis or cystic, heterogeneous parenchyma in pre-enhancement scanning, pseudocapsule, wedge shape sign, degree of CT attenuation in pre-enhancement scanning, enhancement in three scanning phases, enhancement pattern, ESR 1, and ESR 2 showed a statistically significant difference between the two groups (P <0.05).

**Table 1:** Comparison of Characteristics Between Patients with ccRCC and RAML-wvf.

	Patients with ccRCC (n = 93)	Patients with RAML-wvf (n = 49)	P
Age	57 (33–84)	54 (26–90)	0.267
Gender			<0.001
Male	67 (72.0)	18 (36.7)	
Female	26 (28.0)	31 (63.3)	
Amount			1
Single	88 (94.6)	46 (93.9)	
Multiple	5 (5.4)	3 (6.1)	
Growth pattern			0.163
Pattern A	20 (21.5)	13 (26.5)	
Pattern B	71 (76.3)	32 (65.3)	
Pattern C	2 (2.2)	4 (8.2)	
Pattern D	0 (0)	0 (0)	
Contour			0.175
Regular	69 (74.2)	31 (63.3)	
Irregular	24 (25.8)	18 (36.7)	
Edge			0.184
Blurred	35 (37.6)	13 (26.5)	
Clear	58 (62.4)	36 (73.5)	
Wedge shape sign			<0.001
No	82 (88.2)	29 (59.2)	
Yes	11 (11.8)	20 (40.8)	
Round tumor-kidney interface			0.353
No	76 (81.7)	43 (87.8)	
Yes	17 (18.3)	6 (12.2)	
Pseudocapsule			<0.001
No	25 (26.9)	45 (91.8)	
Yes	68 (73.1)	4 (8.2)	
Necrosis or cystic			<0.001
No	30 (32.3)	40 (81.6)	
Yes	63 (67.7)	9 (18.4)	
Calcification			1
No	90 (96.8)	46 (95.8)	
Yes	3 (3.2)	2 (3.2)	
Heterogeneous tumor parenchyma in pre-enhancement scanning			0.002
No	74 (79.6)	48 (98.0)	
Yes	19 (20.4)	1 (2.0)	
Degree of CT attenuation in pre-enhancement scanning			<0.001
Not-high	63 (67.7)	3 (6.1)	
High	30 (32.3)	46 (93.9)	
Enhancement in pre-enhancement scanning			<0.001
Homogeneous	26 (28.0)	34 (69.4)	
Heterogeneous	67 (72.0)	15 (30.6)	
Enhancement in CMP			<0.001
Homogeneous	7 (7.5)	35 (71.4)	
Heterogeneous	86 (92.5)	14 (28.6)	
Enhancement in NP			<0.001
Homogeneous	18 (19.4)	41 (83.7)	
Heterogeneous	75 (80.6)	8 (16.3)	
ESR 1			<0.001
<1–1	44 (47.3)	43 (87.8)	
≥1	49 (52.7)	6(12.2)	
ESR 2			0.011
<1	78 (83.9)	48 (98.0)	
≥1	15 (16.1)	1(2.0)	
Enhancement pattern			<0.001
Fast-in-fast-out	76 (81.7)	20 (40.8)	
Fast-in-slow-out	10 (10.8)	21 (42.9)	
Persistent enhancement	7 (7.5)	8 (16.3)	

### Establishment of the Primary Model

In the univariate analysis, necrosis or cystic, heterogeneous parenchyma in pre-enhancement scanning, pseudocapsule, absence of wedge shape sign, non-high CT attenuation in pre-enhancement scanning, enhancement in three scanning phases (heterogeneous), enhancement pattern, ESR 1, and ESR 2 were significantly associated with ccRCC compared with RAML-wvf

It was confirmed that there was no multicollinearity among these factors by checking tolerance (>0.1) and variance inflation factor (VIF <10) before they were obtained in multivariate analysis. Multivariate analysis demonstrated four independent risk factors for distinguishing ccRCC: pseudocapsule, heterogeneous parenchyma in pre-enhancement scanning, non-high attenuation in pre-enhancement scanning, and heterogeneous enhancement in CMP (**Table 2**), which would be adopted to develop the distinguishing scoring system. The Hosmer-Lemeshow goodness-of-fit test indicates good calibration of this primary predictive model (P = 0.365, >0.05). The ROC (0.977; 95% CI, 0.954–1.000; P = 0.012) shows a good result.

**Table 2:** Predictors of Distinguishing scoring system of ccRCC.

	Univariate analysis	P	HR	Multivariate analysis	β	Score
	P			95% CI		
Wedge shape sign (no)	<0.001	0.069				
Pseudocapsule (yes)	<0.001	0.04	10.824	2.133–54.922	2.382	2
Necrosis or cystic (yes)	<0.001	0.216				
Heterogeneous tumor parenchyma in pre-enhancement scanning (yes)	0.016	0.049	17.513	1.276–240.377	2.863	2
Degree of CT attenuation in pre-enhancement scanning	<0.001	<0.001				
Not-high			232.451	15.118–3574.181	5.449	4
High						
Density pattern in pre-enhancement scanning (heterogeneous)	<0.001	0.71				
Density pattern in CMP (heterogeneous)	<0.001	0.01	60.25	4.722–768.737	4.099	3
Density pattern in NP (heterogeneous)	<0.001	0.348				
ESR 1 (<1)	<0.001	0.957				
ESR 2 (≥1)	0.034	0.059				
Enhancement pattern	<0.001					
Fast-in-fast-out		0.295				
Fast-in-slow-out		0.137				
Persistent enhancement		0.295				

### Establishment of the Scoring System

**Figure 2:**
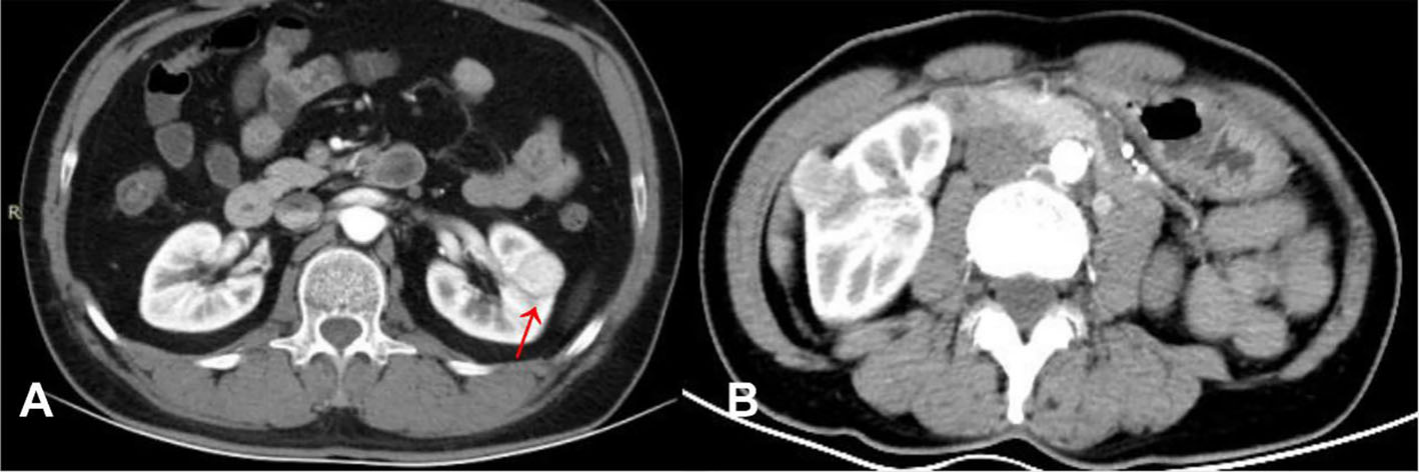
**(A)** ccRCC in a 46-year-old male, post-contrast image depicted an unenhanced arc area between the lesion and renal parenchyma (arrow). **(B)** RAML-wvf in a 55-year-old male, there is no pseudocapsule sign that could be seen.

**Figure 3:**
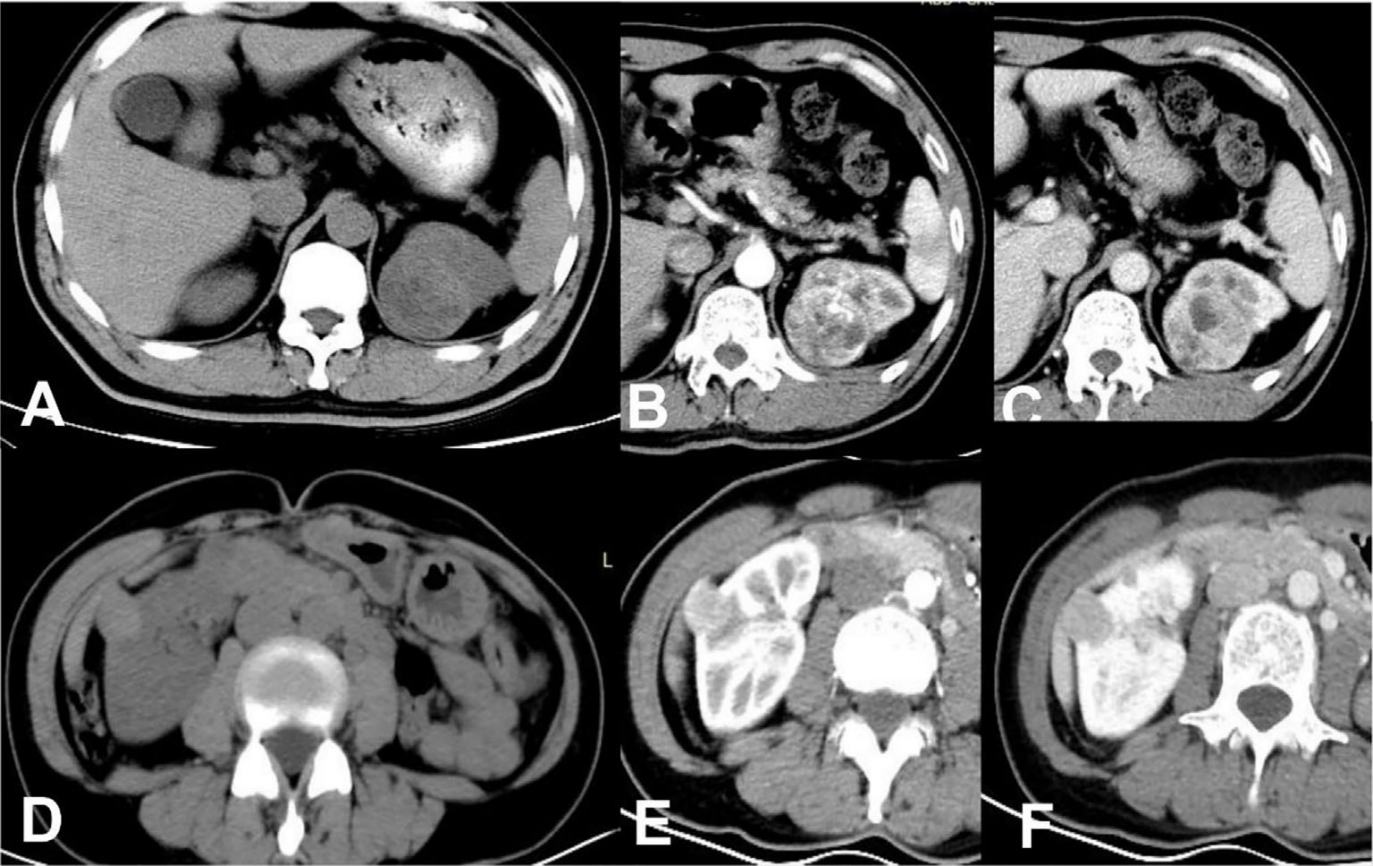
ccRCC in a 48-year-old male, which presented not-high attenuation in plain scanning **(A)**, the attenuation of the mass parenchyma that could be enhanced was mixed. Besides, it was heterogeneous enhanced in CMP **(B)** or NP **(C)**, thus a score of 9 was assigned in this patient. RAML-wvf in a 61-year-old female showed homogeneous high attenuation in plain scanning **(D)** and homogeneous enhancement pattern in CMP **(E)** or NP **(F)**, thus a score of 0 was assigned in this patient and presented one of the minimum values in this model.

**Figure 4:**
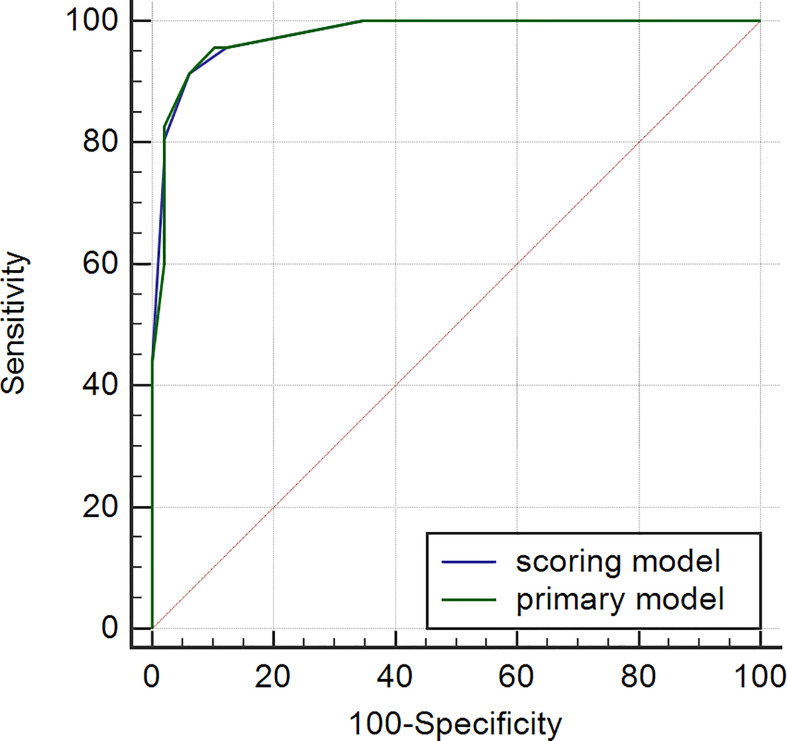
ROC of primary and scoring model.

We assigned risk scores relative to the regression coefficient of each variable that showed statistical significance in the multivariate analysis (**Table 2**): 2 points for tumors having a pseudocapsule (**Figure 2**); 2 points for heterogeneous tumor parenchyma in pre-enhancement scanning (**Figure 3**); 4 points for non-high attenuation in pre-enhancement scanning (**Figure 3**); and 3 points for heterogeneous enhancement in CMP (**Figure 3**). In the distinguishing scoring system, the total score was calculated by summing the individual scores corresponding to the related variables, which produced scores ranging from 0 to 11 points. The Hosmer-Lemeshow goodness-of-fit test indicated good calibration of this scoring model (P = 0.918). The prediction accuracy of this distinguishing scoring system, measured by ROC, was 0.978 (95% CI, 0.956–0.999; P = 0.011). This is similar to the primary model, presenting good distinguishing power for ccRCC, with a sensitivity of 91.4% and a specificity of 93.9% that can be achieved when using 4.5 points as the cutoff value. A comparison of ROC showed no statistical difference between the two models (P = 0.651) (**Figure 4**), which indicates that the distinguishing scoring system has made full use of the primary predictive model.

To provide further convenience for radiologists, we divided the final scores into three ranges: 0 to <2 points; 2–4 points; and >4 to ≤11 points. Patients with ccRCC among the three ranges significantly increased with increasing scores (**Table 3**).

**Table 3:** Patients with ccRCC among three groups in Training cohort and validation cohort.

Score groups	Number of patients with ccRCC		Total Number		Diagnostic probability of ccRCC	
	Training cohort	Validation cohort	Training cohort	Validation cohort	Training cohort	Validation cohort
0 to <2 points	0	0	31	18	0%	0%
2–4 points	8	4	23	10	34.8%	40%
>4 to ≤11 points	85	26	88	32	96.6%	81.25%

Internal validation of the distinguishing scoring system showed good results. The validation cohort included 30 patients with ccRCC and 30 patients with RAML-wvf. Among the scoring ranges, ccRCC patients were 0/18 (0%) of the first (0 to <2 points) range; 4/10 (40%) of second range (2–4 points); and 26/32 (81.25%) of the last range (>4 to ≤11 points) (**Table 3**). The prediction accuracy measured by ROC was 0.922 (95% CI, 0.854–0.991, P = 0.035).

## Discussion

CT is the first-line imaging method used to evaluate renal masses in clinical practice. Patients with RAML-wvf can avoid unnecessary surgery for suspected RCC when an accurate diagnosis is determined preoperatively by CT. Previous studies have proposed specific CT imaging characteristics for differentiating between RAML-wvf and ccRCC. Yang et al. (25) reported that being female, an angular interface, a hypodense rim, homogeneity, and high, unenhanced attenuation were useful characteristics that suggest RAML-wvf However, as described above, using these characteristics or searching for hidden fat tissue is not clinically convenient. Quantitative methods have been reported recently, Hodgdon et al. and Yan et al. (19, 20)proposed that CT texture analysis can quantitatively distinguish between RAML-wvf and ccRCC at three phases with nonlinear discriminant analysis. Lee et al. (26) proposed a texture-based classification system using a three-feature selection method and four-feature classifiers. Nie et al. (21) developed a radiomics nomogram that incorporates a radiomics signature and clinical factors for preoperative differentiation between RAML-wvf and RCC. However, for general radiologists, these technologies may need to be verified and perfected by big data before they can be mature and widely used in clinical practice. We have developed a reliable, convenient-to-use, scoring system consisting of four evaluable factors for discriminating between ccRCC and RAML-wvf based using CT. The simple score system and high accuracy are important strengths of our model, it is simple to use and can be verified by the users including clinicians and radiologists, which make it easier to be widely used.

Among the three ranges in the scoring system, there were no patients with ccRCC in either the training cohort or validation cohort for the first range (0 to <2 points). This indicates that RAML-wvf is more likely to be diagnosed when none of the factors is observed. In the third range (>4 to ≤11 points), 96.6% of patients had ccRCC (81.25% in the validation cohort), indicating that ccRCC is more likely to be diagnosed when more than two critical factors are observed.

Four independent risk factors are included in the system: 1) presence of a pseudocapsule, 2) a heterogeneous tumor parenchyma in pre-enhancement scanning, 3) a non-high CT attenuation in pre-enhancement scanning, and 4) a heterogeneous enhancement in CMP. In addition, women were found to be more likely to have RAML-wvf compared to ccRCC, which is consistent with previous results (11). However, since the desired scoring system is based on CT findings, the patient’s sex was not incorporated into the model. A round tumor-kidney interface and calcification are reported as meaningful phenomena previously (27), but showed no statistical significance in this study. Wedge-shaped signs and necrotic or cystic lesions showed a significant difference between RAML-wvf and ccRCC, according to Ma et al. (28), but they did not show statistical significance in multivariate analysis in this study.

Among the independent risk factors, a non-high CT attenuation of the tumor in pre-enhancement scanning is the predominant factor, which means the tumor attenuation is lower than the attenuation of renal parenchyma. This indicates that RAML-wvf more often presents with high-CT attenuation in pre-enhancement scanning, according to previous reports that found hyperattenuating presentation was a useful method for discriminating between RAML-wvf and ccRCC (12–14). RAML-wvf tends to present homogeneous enhancement after contrast agent administration compared to RCC (12, 13, 15), which is consistent with our research findings (69.4%, 71.4%, 83.7%, respectively, in three enhancement phases). The heterogeneous enhancement pattern is more suggestive of RCC in terms of HR (HR, 60.25; 95% CI, 4.722–768.737), and heterogeneity in this study was defined as the difference between the highest and lowest attenuations being more than 30% of the highest value. Heterogeneous tumor parenchyma in pre-enhancement scanning is also a meaningful factor in terms of HR (17.513; 95% CI, 1.276–240.377), which defined as the parenchyma mass that could be enhanced was mixed. This heterogeneous appearance in unenhanced and enhanced scanning may be due to the fact that ccRCC is an adenocarcinoma derived from renal tubular epithelial cells, often with hemorrhage, necrosis, and cystic, growing rapidly, and presenting a high degree of malignancy. The pseudocapsule is one of the indications of malignancy (29), composed mainly of a fibrous pseudocapsule and compressed renal parenchyma, which presents as an unenhanced arc area between the lesion and renal parenchyma. Yamashita et al. and Sung et al. reported, respectively, that the pseudocapsule sign was found in 66% and 90% of small RCCs, and was also observed in 0% to 10% of RAML-wvf (28, 30, 31). In this study, 73.1% of patients with ccRCC presented with the pseudocapsule sign (8.2% in RAML-wvf), and the HR of this sign was 10.824 (95% CI, 2.133–54.922). RAML-wvf, presenting the biological behavior of benign tumors in most cases, grows as a non-invasive pattern and exerts less pressure on the adjacent renal tissues, which may result in a low probability of pseudocapsule formation.

This study has several limitations. First, there may be an inherent selection bias due to the retrospective study design. Second, although we have collected more cases than previous reports, the total sample size was small, mainly because of the low clinical incidence of RAML-wvf, which may increase the risk of overfitting. In addition, the prediction accuracy of this scoring system in the validation cohort was somewhat lower, compared to that in the training cohort, which might be related to biases caused by the relatively small sample size of the validation cohort. A further prospective cohort with a larger sample size is strongly warranted to validate our diagnostic scoring system.

In conclusion, this study investigated risk characteristics of CT features and built a convenient-to-use scoring system incorporating the four most meaningful factors: pseudocapsule, a heterogeneous tumor parenchyma in pre-enhancement scanning, non-high CT attenuation in pre-enhancement scanning, and heterogeneous enhancement in CMP. This scoring system could be valuable for discriminating ccRCC from RAML-wvf in clinical practice, although a further prospective cohort with a larger sample size will be required to confirm these results.

## Data Availability Statement

The original contributions presented in the study are included in the article/supplementary material. Further inquiries can be directed to the corresponding author.

## Ethics Statement

This study was approved by the local institutional review board from The Second Affiliated Hospital of Zhejiang University and informed consent was waived according to the retrospective design.

## Author Contributions

The work reported in the above for publications has been done by all authors. X-JW contributed to data analysis and manuscript editing. B-QQ collected the data of patients and J-XX supported. J-PZ, Y-FL, Y-YM, and H-QW helped in images analysis. Q-MZ helped in manuscript preparation. And R-SY contributed to the supervision of the whole process. All authors contributed to the article and approved the submitted version.

## Conflict of Interest

The authors declare that the research was conducted in the absence of any commercial or financial relationships that could be construed as a potential conflict of interest.

## Abbreviations

ccRCC, clear cell renal cell carcinoma; RAML-wvf, renal angiomyolipoma without visible fat; ROC, receiver operating characteristic; CMP, corticomedullary phase; NP, nephrographic phase; EP, excretory phase.
